# BET inhibitor JQ1 enhances anti-tumor immunity and synergizes with PD-1 blockade in CRC

**DOI:** 10.7150/jca.69375

**Published:** 2022-03-28

**Authors:** Huijin Wang, Guangyao Liu, Xinghan Jin, Shenglei Song, Songyao Chen, Peiqing Zhou, Huan Li, Jianming Liang, Bo Li, Changhua Zhang, Yulong He

**Affiliations:** 1 Digestive Diseases Center, The Seventh Affiliated Hospital of Sun Yat-Sen University, No.628, Zhenyuan Road, Guangming District, Shenzhen, 518107, China; 2 Department of Gastrointestinal Surgery, The First Affiliated Hospital, Sun Yat-Sen University, 58 Zhongshan 2nd Road, Guangzhou, Guangdong, 510080, China

**Keywords:** Colorectal cancer, JQ1, immunotherapy, immunosuppression.

## Abstract

Most colorectal cancer (CRC) patients are insensitive to immune checkpoint inhibitors (ICIs) due to the immunosuppressive tumor microenvironment (TME). Epigenetic factors such as the bromo-and extraterminal domain (BET) family proteins may be responsible for the immunosuppressive microenvironment. Previous studies have shown that inhibitors of BET family proteins have the potential to remodel the immunosuppressive TME. However, data on the role of BET inhibitors in immune microenvironment in CRC remains unclear. Here, we evaluated the immunoregulatory role of JQ1, a BET inhibitor, in CRC. Transcriptome sequencing data showed that JQ1 decreased *CD274* expression and increased *H2Kb* expression in MC38 cells. Flow cytometry assays demonstrated that JQ1 decreased cell-surface PD-L1 expression in MC38 and HCT116 cells. Moreover, JQ1 significantly increased cell-surface expression of major histocompatibility complex class I (MHC-I) in MC38 cells and HCT116 cells. Antigen-specific cytotoxic T lymphocytes (CTLs) assay demonstrated that JQ1 enhanced the MHC-I-mediated cytotoxicity of CTLs. Mouse colon cancer cell line MC38 was used to establish the syngeneic mouse tumor model. Compared with the control, JQ1 significantly inhibited tumor growth and prolonged the overall survival of the mice. Besides, JQ1 did not only inhibit tumor growth by enhancing anti-tumor immunity, but also promoted the anti-tumor effect of PD-1 antibody. In addition, our data showed that JQ1 reduced infiltration of intratumoral regulatory T cells (Treg), thus remodeling the immunosuppressive TME. Taken together, these results highlight a new approach that enhances anti-PD-1 sensitivity in CRC.

## Introduction

Despite advances in treatment, colorectal cancer (CRC) remains the second leading cause of cancer-related deaths worldwide 1. Programmed cell death ligand 1 (PD-L1), which is expressed on the surface of cancer cells, binds to programmed cell death 1 (PD-1) and inhibits T cell activity, which results in immunosuppression 2, 3. Immunotherapy targeting the PD-L1/PD-1 immune checkpoint has shown significant efficacy in solid tumors, including melanoma and non-small-cell lung cancer (NSCLC) 4, 5. However, PD-L1/PD-1 blockade is effective in only CRC patients with mismatch repair-deficient and microsatellite instability-high (dMMR-MSI-H) phenotypes 6. Most CRC patients are resistant to immune checkpoint inhibitors (ICIs), probably due to low tumor mutation burden and immunosuppressive TME 7, 8.

In addition to the PD-L1-mediated immunosuppression, suppression of MHC-I-mediated tumor antigen presentation is another key factor in immune escape 9. CD8+ T cells are activated by recognition of specific antigens presented by MHC-I. Previous data has shown that downregulation of MHC-I molecules in cancer leads to dysfunction and even exhaustion of cytotoxic T cells 10. Therefore, expression of MHC-I in cancer cells plays a crucial role in anti-tumor immunity 11. Besides, previous studies have demonstrated that loss of MHC-I molecules in cancer reduces sensitivity to PD-L1/PD-1 blockade 12. Correspondingly, upregulation of MHC-I molecules synergizes with PD-L1/PD-1 blockade to enhance anti-tumor activity 13. Several studies have confirmed loss of MHC-I molecules in CRC 14, 15. Thus, we hypothesized that the loss of MHC-I molecules mediates the suppression of anti-tumor immunity in CRC. Upregulation of MHC-I may be a viable strategy to enhance anti-tumor immunity in CRC.

Epigenetic modifications regulate gene expression without altering DNA sequences 16. The epigenetic alterations are important drivers of cancer development and are associated with tumor immune escape 17, 18. Some epigenetic drugs have been shown to remodel the TME and reverse immunosuppression in tumor-bearing animals 19, 20. In addition, epigenetic drugs may strengthen the anti-tumor effects of ICIs 21. The bromo- and extraterminal domain (BET) family proteins are epigenetic factors that regulate gene transcription through reading chromatin histone acetylation 22. For instance, BRD4 is a BET family protein that affects tumor immunity by regulating transcription of the *CD274* (PD-L1) gene in certain cancer cells. JQ1, a BRD4 inhibitor, downregulates the expression of PD-L1, which leads to reduced immunosuppression 23-25. Besides, JQ1 inhibits tumor growth by enhancing anti-tumor immunity. Moreover, JQ1 facilitates anti-tumor effects of ICIs. For instance, combination of JQ1 and PD-1 blockade enhances the anti-tumor effect in Kras-mutant NSCLC 26. JQ1 has also been shown to potentiate anti-tumor immunity with anti-PD-L1 therapy in hepatocellular carcinoma 27.

Since CRC is insensitive to immunotherapy due to the immunosuppressive TME, BET inhibitors could reverse the immunosuppressive TME. However, the role of BET inhibitors in the immune microenvironment of CRC remains unclear. Here, we evaluated the immunoregulatory role of the BET inhibitor, JQ1, in CRC.

## Materials and methods

### Cell lines and lentivirus infection

Mouse colon cancer cell line MC38 and human CRC cell lines HCT116, DLD1, LoVo, LS174T were purchased from the Cell Bank of the Chinese Academy of Sciences. MC38 *BRD4* knockdown cell line and MC38-OVA cell line were generated by lentivirus infection. Knockdown lentivirus of* BRD4* was acquired from GeneCopoeia (China). The sequence for mouse *BRD4* shRNA was 5ʹ-GGTACCAAACACAACTCAAGC-3ʹ; The sequence for human *BRD4* shRNA was 5ʹ-GCTCAAGACACTATGGAAACA-3ʹ. Overexpression lentivirus of ovalbumin was obtained from Syngentech (Beijing, China). Cells were grown in RPMI 1640 medium supplemented with 10% fetal bovine serum (FBS), 1% penicillin and streptomycin.

### Colony formation assay

500 MC38 cells per well were cultured in 6-well plates containing RPMI 1640 medium with 1 μM or 0.1 μM JQ1. The medium was changed every 3 days for 14 days. Cells were washed with PBS and fixed with 4% paraformaldehyde*.* The cells were stained with 0.1% crystal violet for 30 minutes prior to analysis.

### Western blot analysis

We resolved the protein samples in SDS-PAGE. The samples were then transferred into 0.45-μm polyvinylidene difluoride (PVDF) membranes. The membranes were blocked with 5% bovine serum albumin (BSA) at room temperature for 1 hour and then incubated with primary antibodies overnight at 4°C. Thereafter, the blots were incubated with secondary antibodies conjugated with horseradish peroxidase (HRP). The HRP signal for protein expression was detected by electrochemiluminescence (ECL). Target protein signals were quantified and analyzed by ImageJ software. The primary antibodies included: anti-BRD4 antibody: catalog number ab128874, 1:1000, Abcam; and anti-ovalbumin antibody: catalog number ab181688, 1:1000, Abcam.

### Quantitative real-time PCR and RNA sequencing

TRIzol (Invitrogen, USA) was used to extract total RNA from the cells. cDNA was then synthesized from 1 µg of the RNA using the Evo M-MLV RT Premix for qPCR Kit (Accurate Biology, China) using oligo (dT) primers. Quantitative real-time PCR (qPCR) was performed using a SYBR Green Premix Pro Taq HS qPCR Kit (Accurate Biology, China), following the manufacturer's instructions. RNA-sequencing was evaluated by Lc-bio Technologies co., ltd. (Hangzhou, China). In Brief, the MC38 cells were treated with the JQ1 inhibitor for 48 hours. TRIzol (Invitrogen, USA) was used to extract total RNA (Invitrogen, USA), which was then purified and used to synthesize cDNA. The cDNA libraries were sequenced on an Illumina Novaseq™ 6000 (Lc-bio Technologies co., ltd. Hangzhou, China).

### Antigen-specific CTLs assay

MC38-OVA cells were incubated with JQ1 for 48 hours. The cells were then labeled with PKH26 (MINI26, Sigma, USA) according to the manufacturer's instructions. After removal of residual PKH26, the cells were incubated with 100 ng/ml Ovalbumin (OVA) peptide (AnaSpec, USA) for 12 hours. We harvested and washed the cells, and then plated them at a density of 5×10^6^ cells /cm^2^, followed by incubation for 6 hours. OT-I transgenic mice were obtained from The Jackson Laboratory (003831, USA). The mice were sacrificed and then spleens and lymph nodes were prepared into single-cell suspensions. The cells were cultured in complete medium, supplemented with 10 ng/ml murine IL-2 (PeproTech, USA) for an additional 24 hours. CD8+ T cells were enriched using mouse CD8a+ T cell magnetic microbeads (Miltenyi Biotec, Germany). The CD8+ T cells were incubated in a medium containing 100 ng/ml OVA peptide for 24 hours. After washing with PBS to remove the OVA peptide, the cells were expanded in a medium containing murine 10 ng/ml IL-2 for 48 hours. OT-1 CD8+ T cells were harvested and counted, followed by coculture with MC-38OVA cells, labeled with PKH26 for 12 hours. On the other hand, dead cells were labeled with FVS780 (catalog number 565388, BD). PKH26+FVS780+ double-positive MC38-OVA cells were lysed by CD8+ T cells.

### Animal models and treatments

Animal experiments were approved by the Experimental Animal Care Commission of Shenzhen Top Biotechnology Co.,Ltd (Number: TOP-IACUC-2021-0103). Six- to eight-week old female BALB/c nude mice and C57BL/6 mice were purchased from BesTest Biotechnology company (Zhuhai, China). MC38 cells (1×10^6^) were injected subcutaneously into the right flank of the C57BL/6 or nude mice. The C57BL/6 tumor-bearing mice were randomly divided into four groups (n=5). Tumor size was measured every 2-3 days using electronic vernier calipers. Tumor volumes were calculated using the following formula: longest diameter ×shortest diameter^2^/2. Starting from day 8, mice were injected intraperitoneally with 50 mg/kg JQ1 (5% DMSO in 2-hydroxypropyl-β-cyclodextrin, Selleck, USA) daily for 7 days. For the anti-PD-1 and the combination therapy group, 100 μg of PD-1 antibody (catalog number BP0146, clone RMP1-14, Bioxcell, USA) was intraperitoneally injected on days 9 and 12. The mice were euthanized when they reached endpoints. Humane endpoints were defined as tumors that have 2 cm longest diameter or 2000 mm^3^ volume. The nude mice were randomly distributed into control and experimental group (five mice in each group). The experimental group mice were intraperitoneally injected with 50 mg/kg JQ1 daily. The control group mice were intraperitoneally injected with DMSO. Tumors from both groups were collected and measured on day 8 after treatment.

### Cell harvesting and flow cytometry analysis of Treg cells

Approximately 1×10^6^ MC38 cells were injected into the C57BL/6 mice. The mice were intraperitoneally injected with JQ1 daily for 7 days, starting on day 8 after cell implantation. The tumors were harvested and minced into 1-mm^3^ pieces, and then soaked in a digestion solution containing collagenase P (2 mg/ml, Sigma) and DNase I (50 μg/ml, Sigma) for 30 minutes at 37°C. The digested tumor tissues were filtered through a 40-μm cell strainer to obtain a cell suspension. Dead cells were labeled using Zombie Aqua (Biolegend). The cells were washed with PBS and then blocked with an anti-CD16/32 antibody for 20 minutes on ice. Thereafter, the cells were blocked and incubated with surface antibody for 20 minutes. The cells were then fixed and permeabilized for staining of intracellular antigens. We resuspended the cells in 200 µl of staining buffer (2% FBS in PBS) and then analyzed by flow cytometry (Beckman Coulter Cytoflex LX; Beckman*,* USA). The data were analyzed by FlowJo10 software.

### Antibodies and related reagents for flow cytometry

Human TruStain FcX: clone 93, catalogue number 422302, Biolegend. TruStain FcX (anti-mouse CD16/32) Antibody: clone 93, catalogue number 101320, Biolegend. PE anti-human CD274 (B7-H1, PD-L1) Antibody: clone 29E.2A3, catalogue number 329706, Biolegend. FITC anti-human HLA-ABC Antibody: clone W6/32, catalogue number 311404, Biolegend. PE anti-mouse CD274 (B7-H1, PD-L1) Antibody: clone 10F.9G2, catalogue number 124308, Biolegend. FITC anti-mouse H2Kb Antibody: clone AF6-88.5, catalogue number 116506, Biolegend. FITC anti-mouse CD45: clone 30-F11, catalogue number 103108, Biolegend. Pacific blue anti-mouse CD3ε: clone 145-2c11, catalogue number 100334, Biolegend. Alexa Fluor647 anti-mouse CD4: clone GK1.5, catalogue number 100424, Biolegend. PE anti-mouse Foxp3: clone MF-14, catalogue number 126403, Biolegend. Zombie Aqua Fixable Viability Kit: catalogue number 423102, Biolegend**.** True-Nuclear Transcription Factor Buffer Set: catalogue number 424401, Biolegend. FVS780: catalogue number 565388, BD.

### Statistical analysis

All* in vitro* experiments were conducted in triplicates. Comparison between two independent data sets was performed by two-tailed unpaired t-test. Statistical significance of tumor growth curves was analyzed using paired two-tailed t-test. Differences in the overall survival curves between two groups were analyzed with Log-rank test. Data were expressed as mean ± standard error of the mean (SEM). p< 0.05 indicates statistical significance.

## Results

### JQ1 downregulates PD-L1 expression and upregulates MHC-I expression in CRC

**Figure 1:**
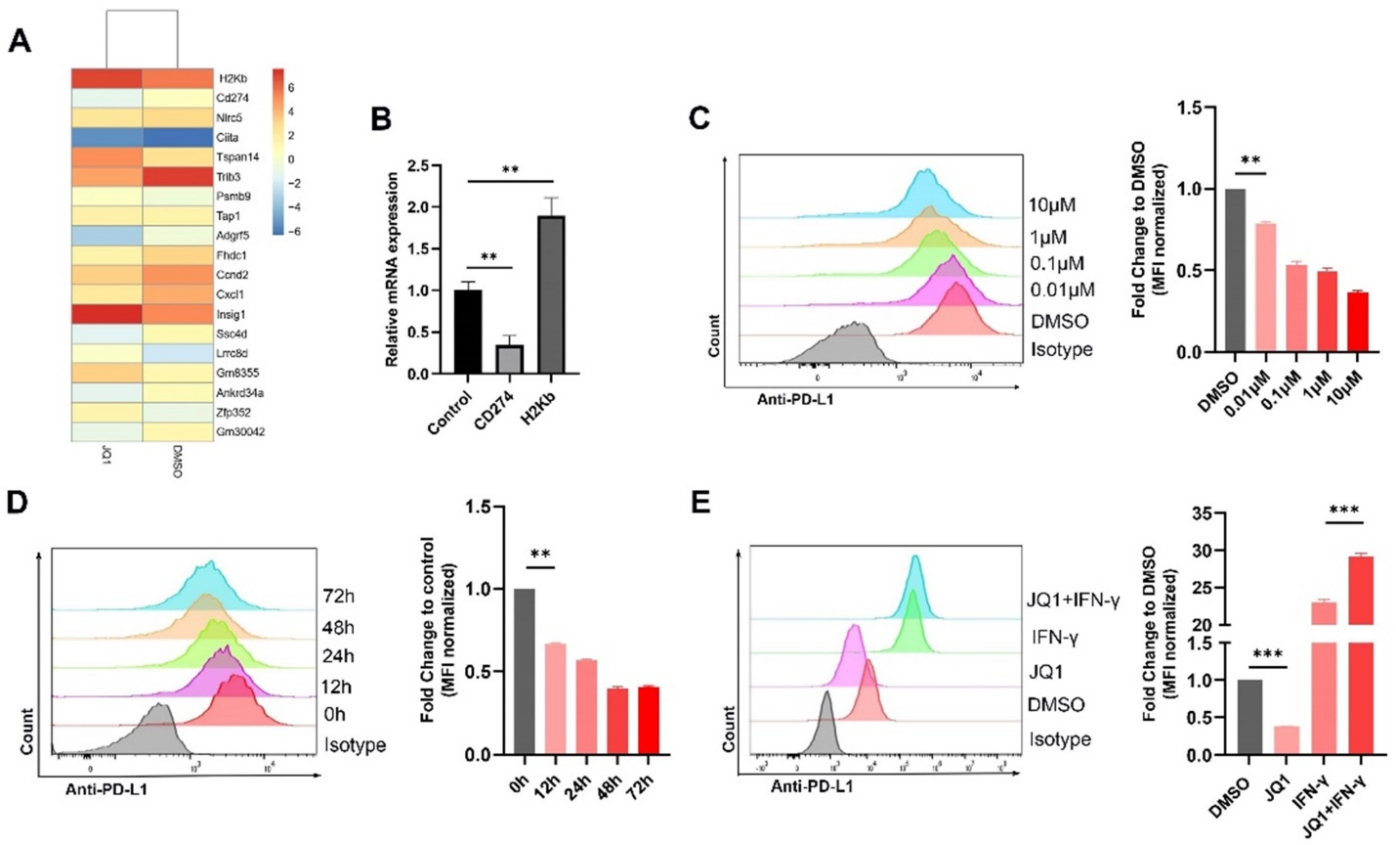
** BET inhibitor JQ1 regulates the expression of PD-L1 in MC38 cells.** (**A**) mRNA sequencing results; (**B**) Confirmation of mRNA sequencing results by qPCR; The surface expression of PD-L1 were determined by flow cytometry: (**C**) Cells were stimulated by different doses of JQ1 for 48 hours; (**D**) Cells were incubated with JQ1 (0.1 μM) for indicated times; (**E**) cells were treated with or without 0.1 μM JQ1 for 48 hours in the absence or presence of IFN-γ (20 ng/ml). *p<0.05, **p<0.01, ***p<0.001, ns, no significant difference.

RNA was extracted and sequenced from the MC38 cells treated with JQ1. The results showed that *CD274* gene was significantly suppressed, while *H2Kb* (*MHC-I*) was upregulated following JQ1 treatment (Fig. 1A). The results were confirmed by qPCR (Fig. 1B). We then employed flow cytometry to analyze the effect of JQ1 on the expression of PD-L1 at indicated doses. The data showed that JQ1 significantly decreased the expression of PD-L1 in a dose-dependent manner (Fig. 1C). To exclude the bias caused by cytotoxicity of JQ1. We selected a drug concentration (0.1 μM), with little effect on cell activity for subsequent experiments based on cell viability assays (Fig. S1A). Use of 0.1 μM JQ1 did not inhibit cell colony formation (Fig. S1B). Thereafter, we assessed PD-L1 expression at indicated times. As expected, JQ1 inhibited PD-L1 expression in a time-dependent manner (Fig. 1D). However, JQ1 increased IFN-γ-induced PD-L1 expression (Fig. 1E). These data demonstrated that there may be multiple mechanisms by which JQ1 regulates PD-L1 expression.

**Figure 2:**
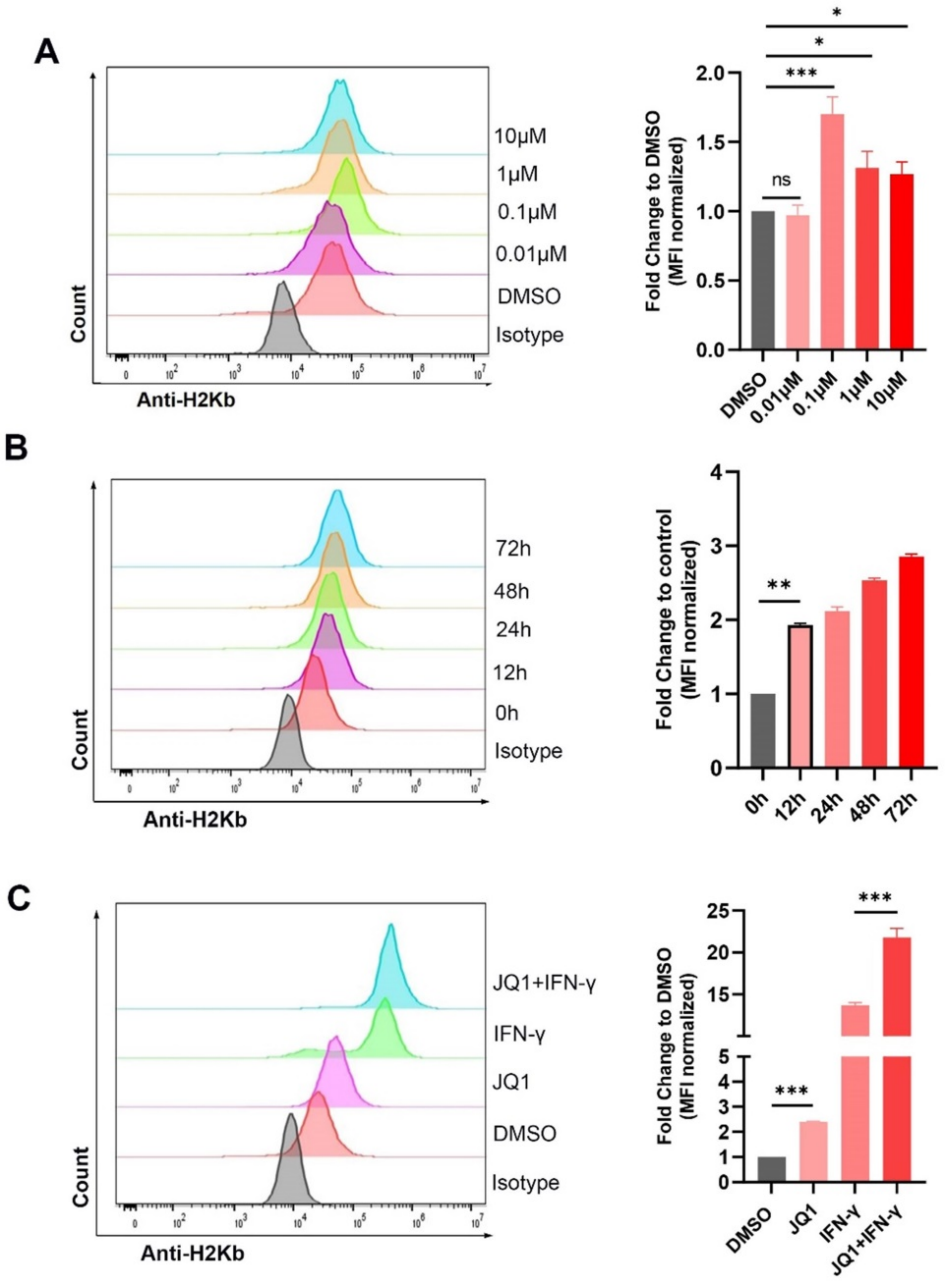
** JQ1 increases the expression of H2Kb in MC38 cells.** The surface expression of H2Kb were evaluated by flow cytometry. (**A**) Cells were stimulated by indicated doses of JQ1 for 48 hours; (**B**) Cells were treated with JQ1 (0.1 μM) for different times; (**C**) cells were incubated with or without 0.1μM JQ1 for 48 hours in the absence *or* presence of IFN-γ (20 ng/ml). *p<0.05, **p<0.01, ***p<0.001, ns, no significant difference.

**Figure 3:**
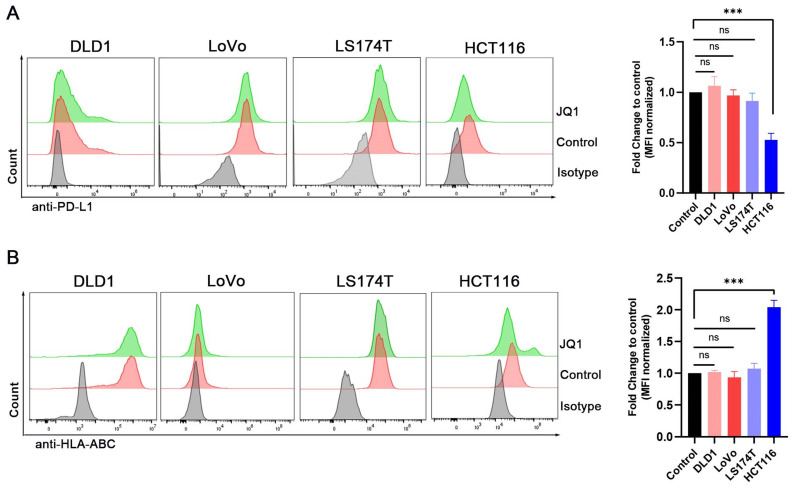
**Expression of PD-L1 and HLA-ABC were examined in the 4 human colorectal cancer cell lines by flow cytometry.** (**A**) The PD-L1 expression of cells treated with 0.1 μM JQ1; (**B**) The HLA-ABC expression of cells treated with 0.1 μM JQ1; *p<0.05, **p<0.01, ***p<0.001, ns, no significant difference.

We also explored regulation of H2Kb by JQ1. JQ1 at 0.1 μM significantly increased the expression of H2Kb in the MC38 cells (Fig. 2A). JQ1 increased the expression of H2Kb in a time-dependent manner (Fig. 2B). In addition, JQ1 facilitated IFN-γ-induced H2Kb expression (Fig. 2C). These results demonstrated that JQ1 promotes both constitutive and IFN-γ-induced expression of H2Kb in the MC38 cells. We then analyzed the expression of PD-L1 and HLA-ABC (MHC-I) in human CRC cell lines treated with JQ1. The data showed that JQ1 significantly decreased the cell surface expression of PD-L1, but increased the cell surface expression of HLA-ABC in HCT116 cells (Fig. 3A,3B).

### JQ1 regulates the expression of PD-L1 and MHC-I through BRD4

**Figure 4:**
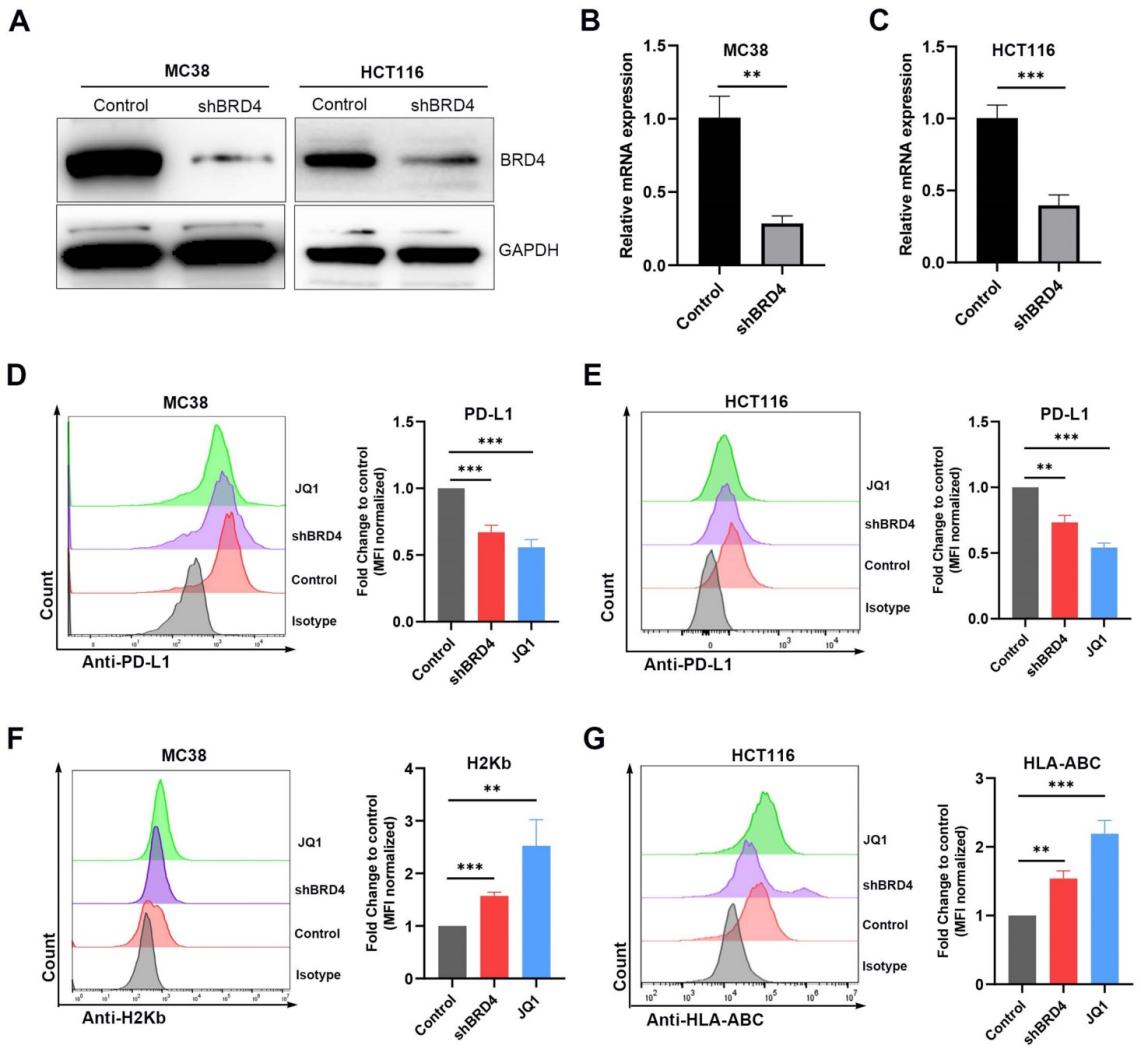
** JQ1 regulates the expression of PD-L1 and MHC-I through BRD4.** The BRD4 shRNA knockdown efficiency was evaluated by western blot (**A**) and qPCR (**B**) (**C**). MC38/HCT116 cells transfected with shBRD4, wild-type cells treated with JQ1 (0.1 μM, 48 hours) were determined for PD-L1 expression (**D**) (**E**) and MHC-I expression (**F**) (**G**) by flow cytometry. *p<0.05, **p<0.01, ***p<0.001, ns, no significant difference.

It has been reported that JQ1 blocks binding of BRD4 to the *CD274* promoter, thereby repressing transcription of *CD274*. To evaluate whether JQ1 affects PD-L1 and MHC-I expression through BRD4, we knocked down the *BRD4* gene in MC38 and HCT116 cells using short hairpin RNAs (shRNA). Western blot (Fig. 4A) and qPCR (Fig. 4B, 4C) analysis demonstrated down regulation of *BRD4*. Compared with the control, silencing of BRD4 reduced PD-L1 expression (Fig. 4D, E). In addition, BRD4 knockdown elevated the expression of MHC-I (Fig. 4F, G). These findings show that BRD4 regulate both PD-L1 and MHC-I genes in CRC.

### JQ1 enhances the cytotoxicity of CTLs by promoting tumor antigen presentation

**Figure 5:**
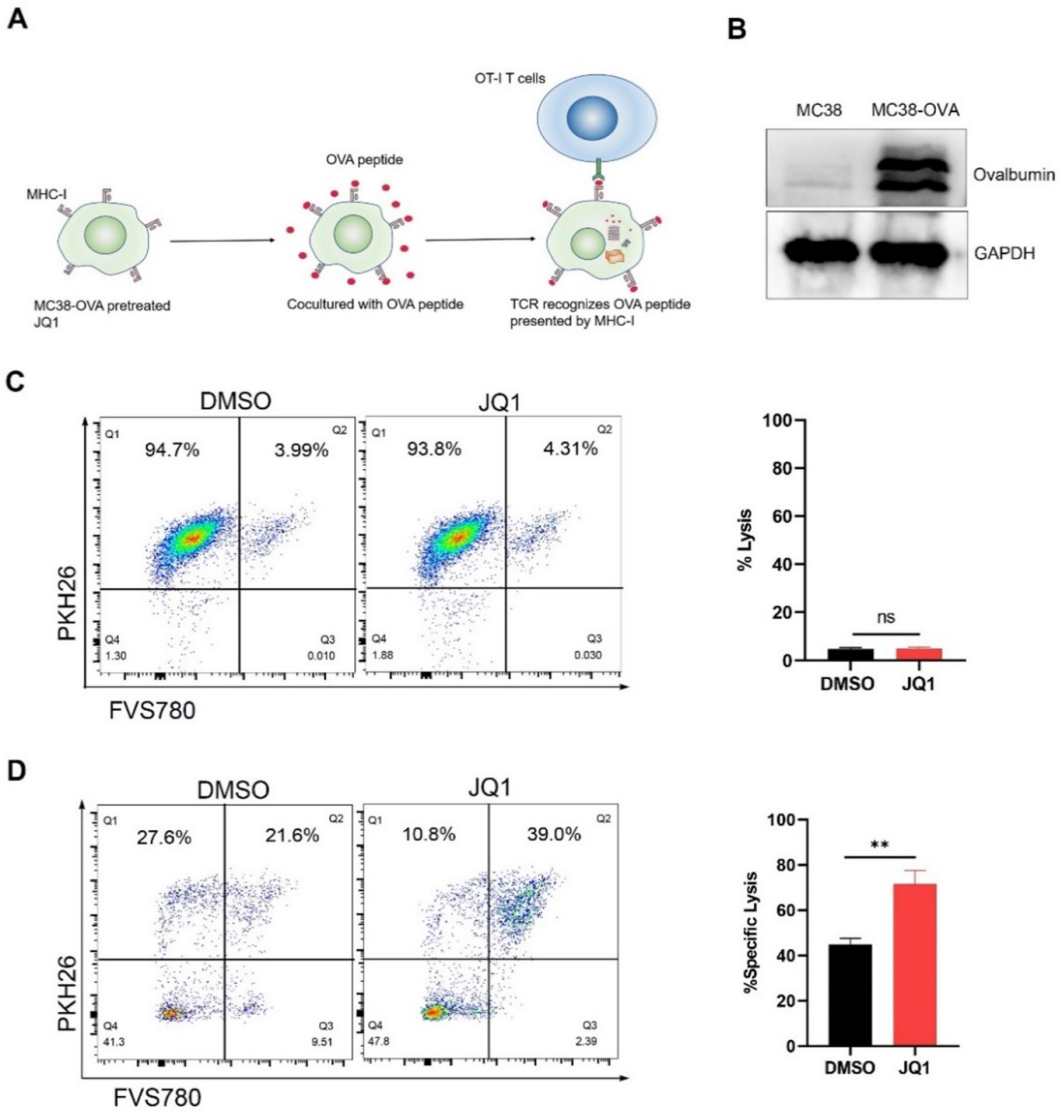
** JQ1 enhances CD8+T cell cytotoxicity by promoting tumor antigen presentation.** (**A**) Illustration of antigen-specific CTLs assay. (**B**) Identification of MC38-OVA cells. (**C**) Percent lysis of MC38-OVA cells pretreated with JQ1 or DMSO in the absence of OT-I CD8+ T cells. (**D**) Specific lysis percentage of MC38-OVA cells pretreated with DMSO or with JQ1 by CD8+ T cells. *p<0.05, **p<0.01, ***p<0.001, ns, no significant difference.

An antigen-specific model was used to evaluate the regulation of CD8+ CTLs cytotoxicity by elevated H2Kb expression. It is well known that CTLs are activated by recognition of antigen/MHC-I complexes. Since ovalbumin (OVA) peptides are not expressed in mammals, they can mimic tumor-specific antigens 28. CD8+ CTLs from OT-I mice express T cell receptors that specifically recognize OVA peptides. Theoretically, JQ1 promotes antigen presentation by increasing the expression of H2Kb, thereby enhancing killing efficiency of CTLs against cancer cells (Fig. 5A).

We used the lentivirus to infect MC38 cells to construct OVA-expression cells. Western blot analysis demonstrated that MC38-OVA cells express OVA (Fig. 5B). To define cell killing by JQ1 toxicity, we compared cytolysis by JQ1 or DMSO in the absence of T cells. Compared with DMSO, incubation with 0.1 μM JQ1 did not enhance cytolysis in the absence of T cells (Fig. 5C). Therefor, 0.1 μM JQ1 treatment had no significant cytotoxicity. For the CTLs assay, MC38-OVA cells were treated with OVA peptides for 24 hours following pretreatment with JQ1. The MC38-OVA cells were then cocultured with CD8+ T cells from OT-I mice. PKH26/FVS780 double-positive MC38-OVA cells were lysed by CD8+ T cells. These data revealed that the proportion of cytolysis in cells treated with JQ1 was notably higher than that in the control group (Fig. 5D). These data demonstrate that JQ1 enhances the killing capacity of CD8+ T cells against MC38-OVA cells. Our results confirm the observation that JQ1 enhances cytotoxicity of CD8+ T cells by promoting MHC-I-mediated antigen presentation.

### JQ1 combined with anti-PD-1 enhances anti-tumor immunity in CRC

**Figure 6:**
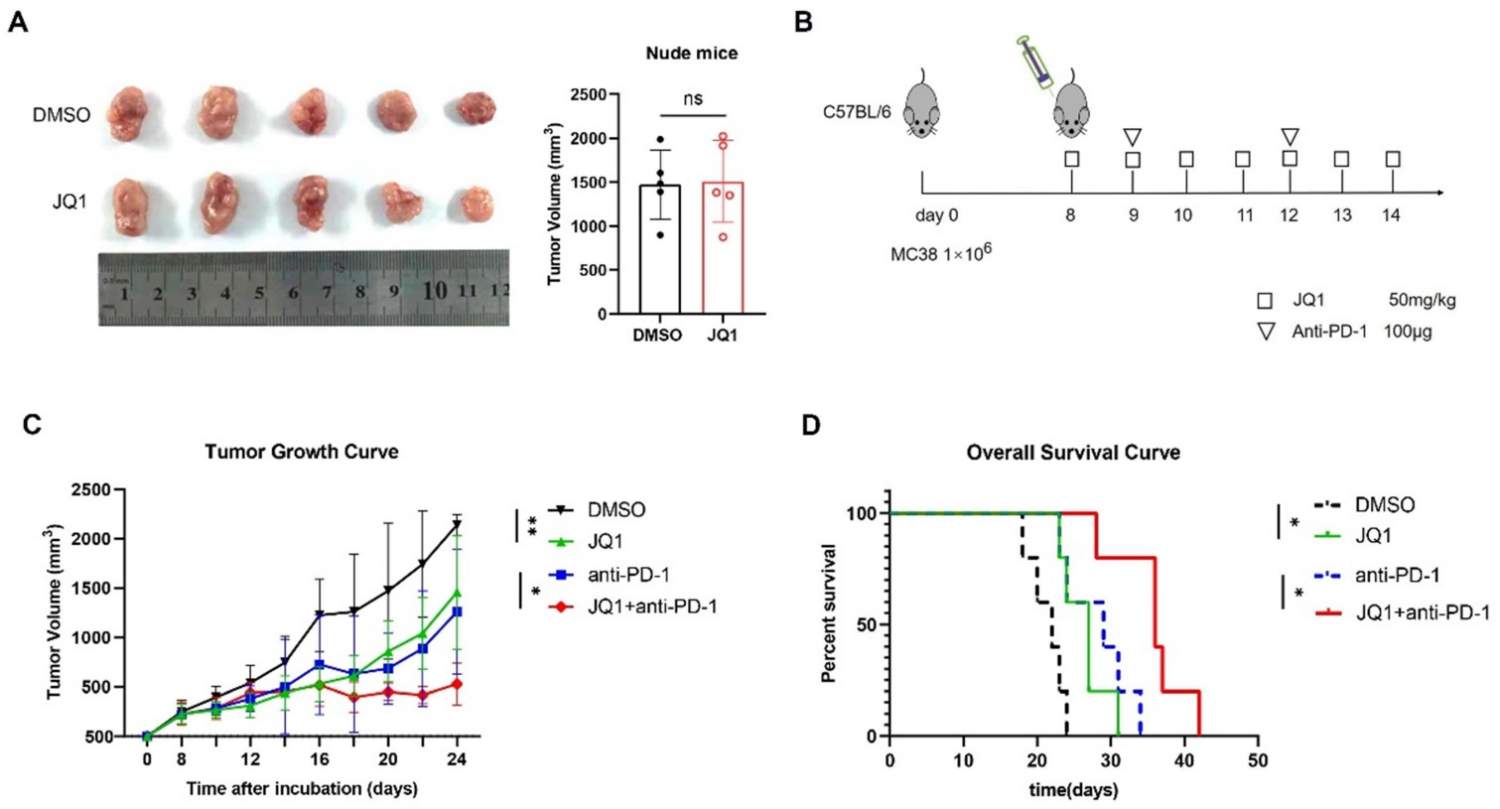
** JQ1 combined with anti-PD-1 enhances anti-tumor immunity *in vivo*.** (**A**) Comparison of tumor volumes from nude mice. n=5 mice per group;** (B)** The animal experimental protocol. n=5 mice per group; **(C)**Tumor growth curve. Statistical significance was analysed using paired two-tailed t-test; **(D)** Overall survival curve. p value tested using Log-rank test. *p<0.05, **p<0.01, ***p<0.001, ns, no significant difference.

Since JQ1 promotes antigen presentation and enhances CD8+ T cell cytotoxicity, we hypothesized that JQ1 could enhance anti-tumor immunity *in vivo*. Moreover, JQ1 upregulates IFN-γ-induced PD-L1 expression, thus showing that JQ1 may synergistically fuel the anti-tumor ability of the PD-1 antibodies. To exclude JQ1 from inhibiting tumor growth through nonadaptive immunity, we established an animal model of T-cell-deficient BALB/c nude mice bearing MC38 cells. Compared with the control, JQ1 did not inhibit tumor growth in nude mice (Fig. 6A). MC38 cells were then subcutaneously injected into C57BL/6 mice to establish a transplantation tumor model with intact immune systems. The mice were then randomly divided into 4 groups: control, JQ1, anti-PD-1, and JQ1+anti-PD-1 (Fig. 6B). As expected, JQ1 treatment markedly inhibited tumor growth compared with the control (Fig. 6C). In comparison with the anti-PD-1 monotherapy, combination of JQ1 and anti-PD-1 exhibited more potent anti-tumor efficacy. Besides, the overall survival of mice treated with JQ1 was prolonged compared to that of the control group (Fig. 6D). Compared with the anti-PD-1 monotherapy, a combination treatment with JQ1 resulted in increased overall survival (Fig. 6D). These results demonstrate that JQ1 enhances anti-tumor immunity in tumor-bearing mice. In addition, JQ1 synergistically augments the anti-tumor capacity of the PD-1 antibody.

**Figure 7:**
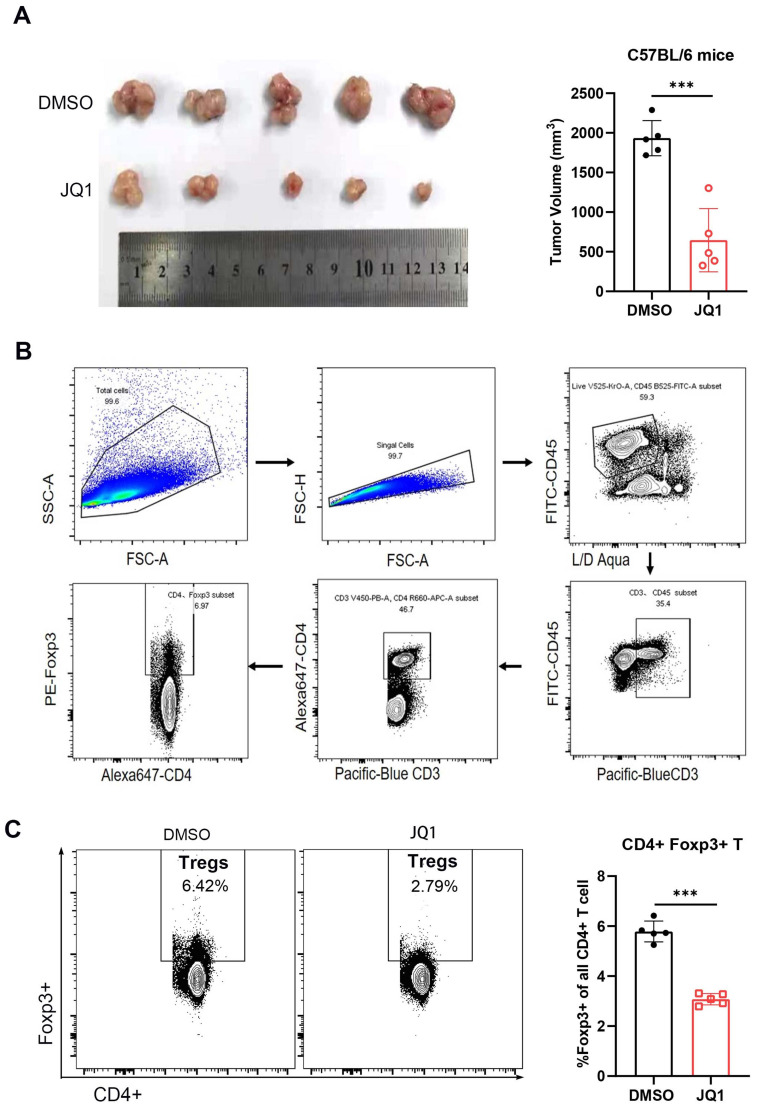
** JQ1 reduces tumor-infiltrating Foxp3+ regulatory T cells.** (**A**) Comparison of tumor volumes from C57BL/6 mice. n=5; **(B)** Flow cytometry gating strategy for analyzing Treg cells; **(C)** Percentage of Foxp3+ Treg cells among CD4+ T cells. n=5. *p<0.05, **p<0.01, ***p<0.001, ns, no significant difference.

Presence of Treg cells is a major cause of immunosuppressive microenvironment in CRC 29. To determine the effect of JQ1 on tumor infiltrating Treg cells, tumor tissues were harvested at the same time point. The results showed that the tumor volume of JQ1-treated mice was significantly smaller compared to that of the control mice (Fig. 7A). The anti-tumor effect of JQ1 was demonstrated again. The cells were then isolated from the tumor tissues for flow cytometric analysis. The data showed that the number of intratumoral Foxp3+ Treg cells in the JQ1 group was less than that in the control group (Fig. 7C). These results demonstrated that JQ1 inhibits the infiltration of Treg cells, leading to enhanced anti-tumor immunity.

## Discussion

Despite FDA approval of ICI drugs for use in dMMR-MSI-H metastatic CRC patients, not all the patients respond to the ICIs 30. Patients with MSI-stable CRC, who account for the majority of CRC cases, do not respond to ICIs treatment 31. Many studies have tried to elucidate the causes of CRC insensitivity to immunotherapy. However, to date, there is no new approach developed to improve immunotherapy outcomes in these patients.

Expression of PD-L1 by cancer cells significantly suppresses the function of T cells, leading to immunosuppression 2. JQ has been shown to inhibit PD-L1 expression in various cancer cells 24, 25. Our data showed that JQ1 inhibits constitutive PD-L1 expression but promotes IFN-γ-induced PD-L1 expression in MC38 cells. Some studies have shown that the level of PD-L1 expression in cancer cells is directly proportional to the effect of PD-1 blockade 32, 33. However, patients with low PD-L1 expression may also respond to PD-1 blockade 31. For CRC, there is no clear relationship between the effect of PD-1 antibody therapy and the expression of PD-L1 34. We observed that the regulation of PD-L1 by JQ1 contributes to enhanced the efficacy of PD-1 blockade. We considered the initial stage of adaptive immunity, during which JQ1 decreases the level of PD-L1 in cancer cells and reduces the inhibition of activated T cells. Activated T cells produce IFN-γ, and JQ1 increases IFN-γ-induced PD-L1 expression in cancer cells, thus enhancing the sensitivity of cancer cells to PD-1 antibody.

MHC-I is a key molecule in tumor antigen presentation. CD8+ T cells recognize only tumor antigens presented by MHC-I. Therefore loss of MHC-I molecules is an important cause of tumor immune escape 9. MHC-I expression in cancer cells does not only predict prognosis, but also therapeutic response to ICIs 12, 35. Upregulation of MHC-I expression partially reverses the immunosuppressive TME 36. In recent years, epigenetic modifications have been shown to play an important role in the regulation of tumor immunity 37. In addition, histone deacetylase inhibitors have been shown to upregulate the expression of MHC-I in cancer cells and to enhance the therapeutic effect of anti-PD-1 on lymphoma 38, 39. JQ1, a BRD4 inhibitor, has been reported to upregulate MHC-I expression in prostate cancer 40. In this study, we found that JQ1 increases MHC-I expression in CRC. However, the mechanism by which JQ1 promotes MHC-I expression remains unclear. It is now clear that JQ1 reduces protein expression by repressing gene transcription 41. We speculate that *MHC-I* is not directly regulated by JQ1 and that there may be a target gene that negatively regulates* MHC-I*. Furthermore, using antigen-specific CTLs assay, we showed that upregulation of MHC-I remarkably strengthened the killing of cancer cells by CD8+ T cells.

We explored the anti-tumor immune effects of JQ1 in the MC38 animal model. The results demonstrated that JQ1 inhibited tumor growth and prolonged survival of the mice. The combination group had better tumor suppression and longer survival compared to the PD-1 antibody monotherapy group. Thus, JQ1 was shown to synergize the anti-tumor effect of PD-1 blockade.

Foxp3+ Treg cells are enriched in the human colorectal cancer TME and are associated with poor prognosis 42. Foxp3+ Treg cells are an immunosuppressive subset of CD4+ T cells 43. Treg cells promote tumor progression by suppressing anti-tumor immunity. This is achieved through consumption of IL-2, secretion of immunosuppressive cytokines, and upregulation of immune checkpoint levels in effector T cells 44-46. These events ultimately impair the function of effector T cells and lead to apoptosis. Treg cells do not only play an important role in tumor progression, but also positively correlates with acquired resistance to ICIs 47. Furthermore, targeting Treg cells contribute to reduction of the acquired resistance to ICIs 48. It has been reported that JQ1 reduces the number of Treg cells in the TME of NSCLC, consequently enhances anti-tumor immunity 49. In this study, we demonstrated that JQ1 significantly reduces intratumoral Treg cells infiltration in MC38 model. Besides, the Treg cells infiltration levels were correlated with tumor progression and poor prognosis. These data demonstrated that JQ1 enhances anti-tumor immunity by remodeling the immunosuppressive microenvironment in CRC.

In conclusion, we revealed that JQ1, a BET inhibitor, regulates PD-L1 and promotes MHCI-I expression in CRC. JQ1 enhances anti-tumor immunity by strengthening CTLs cytotoxicity and reducing Treg cells infiltration. In addition, JQ1 synergizes the anti-tumor effect of PD-1 blockade. These findings highlight a novel approach that improves anti-tumor immunity in CRC and enhances the sensitivity to PD-1 blockade. However, there is need for further studies on the mechanism by which JQ1 regulates MHC-I expression.

## Supplementary Material

Supplementary figure.
