# Supplementary material for: BET inhibitor JQ1 enhances anti-tumor immunity and synergizes with PD-1 blockade in CRC
Source: J Cancer. 2022 Mar 28;13(7):2126–37. doi: 10.7150/jca.69375 (PMC9066214; doi:10.7150/jca.69375)
Supplement: Supplementary file 1 — Supplementary figure. [file jcav13p2126s1.pdf]

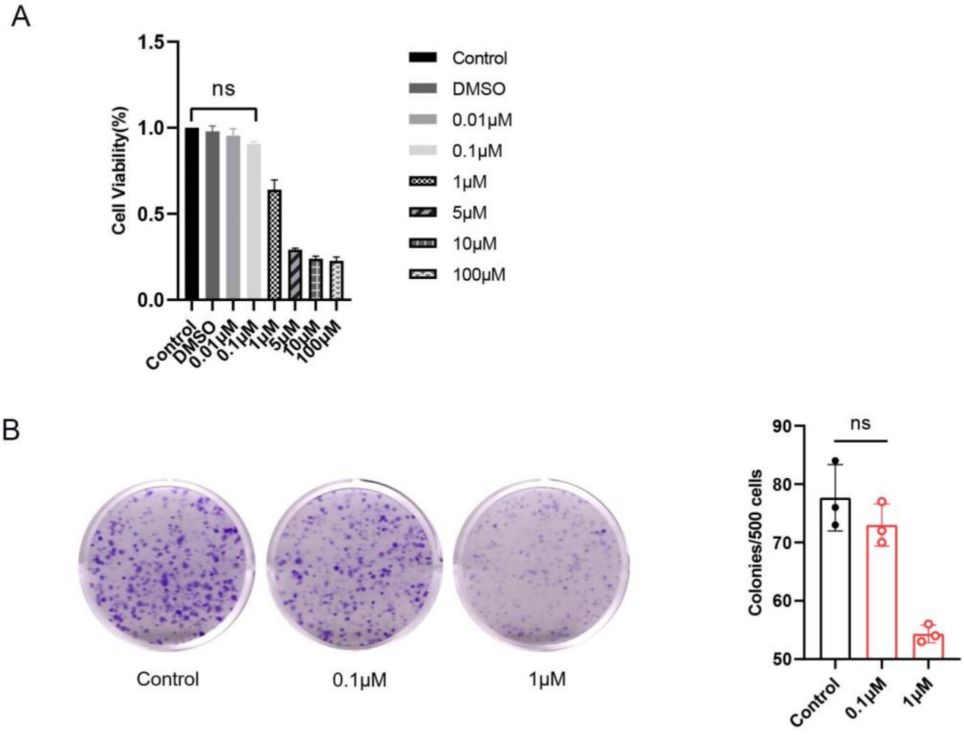

Supplementary figure 1

(A) MC38 cells were treated with indicated doses of JQ1 for 48 h. Cell viability was evaluated by CCK8 assay.

(B) Clone formation ability of MC38 cells pretreated with 0.1 μM JQ1 was determined by clonogenic assay. Ns, no significant difference.
